# Unresolved ER stress restricts in vitro plant cell totipotency

**DOI:** 10.1007/s00299-025-03586-8

**Published:** 2025-09-17

**Authors:** Patricia Corral Martinez, Charlotte Siemons, Michael Schon, Marije Vos, Anneke Horstman, Ruud de Maagd, Jose María Seguí-Simarro, Kim Boutilier

**Affiliations:** 1 Bioscience, Wageningen University & Research, P.O. Box 16, 6700 AA Wageningen, Netherlands; 2 Laboratory of Molecular Biology, Wageningen University & Research, P.O. Box 633, Wageningen, Netherlands; 3 Cell Biology Group, COMAV Institute, Universitat Politècnica de València (UPV), Camino de Vera, s/n, 46022 València, Spain; 4 Enza Zaden, Haling 1, 1602 DB Enkhuizen, Netherlands; 5 Laboratory of Biochemistry, Wageningen University, P.O. Box 8128, 6700 ET Wageningen, Netherlands

**Keywords:** *Brassica napus*, ER stress, Microspore embryogenesis, Totipotency

## Abstract

**Key message:**

Many plant cells can be induced to regenerate in vitro. We show that successful regeneration during microspore-derived embryo culture relies in part on the ability of embryogenic cells to resolve tissue culture-induced ER stress.

**Abstract:**

During *Brassica napus* microspore embryogenesis, the immature male gametophyte is induced by a heat stress treatment to develop into a haploid embryo. Different multicellular embryogenic structures develop in response to heat stress, each with a different potential to complete embryo development. The underlying factors that determine the ability of these initially embryogenic structures to successfully complete embryo development are not known. We show that all embryogenic structures exhibit elements of endoplasmic reticulum (ER) stress, like ER expansion and protein-filled ER cisternae, but that the ER stress response is amplified in embryogenic structures with a low potential to complete embryo development. ER stress was amplified even further by treating heat-stressed cultures with trichostatin A, a histone deacetylase inhibitor epidrug that promotes embryogenic cell formation. Pharmacological treatment of microspore-derived embryo cultures with small molecule modulators of ER stress provided further evidence for the role of ER stress in microspore embryo development. Our results suggest that (1) the inability of certain embryogenic structures to resolve their ER stress responses restricts their ability to complete embryo development, and (2) histone deacetylation enhances microspore embryogenesis in *B. napus*, in part through its activity as an abiotic stress inducer.

**Supplementary Information:**

The online version contains supplementary material available at 10.1007/s00299-025-03586-8.

## Introduction

For most plants, prolonged changes in environmental conditions outside of those experienced during normal plant growth are generally perceived as stressful (Zhang et al. 2023). Conserved abiotic stress responses evolved to ensure survival under adverse conditions by minimizing or reversing the negative effects of stress on development and growth (Zhang et al. 2022). Exposure to abiotic stress can also induce (adaptive) changes in plant development (Kapulnik and Koltai 2016; Samakovli et al. 2020; Pan et al. 2020; Jagadish 2020). An extreme example of this is microspore embryogenesis (ME), where abiotic stress treatments induce in vitro-cultured immature male gametophytes to develop into embryos instead of pollen (Seguí-Simarro et al. 2021).

**Fig. 1:**
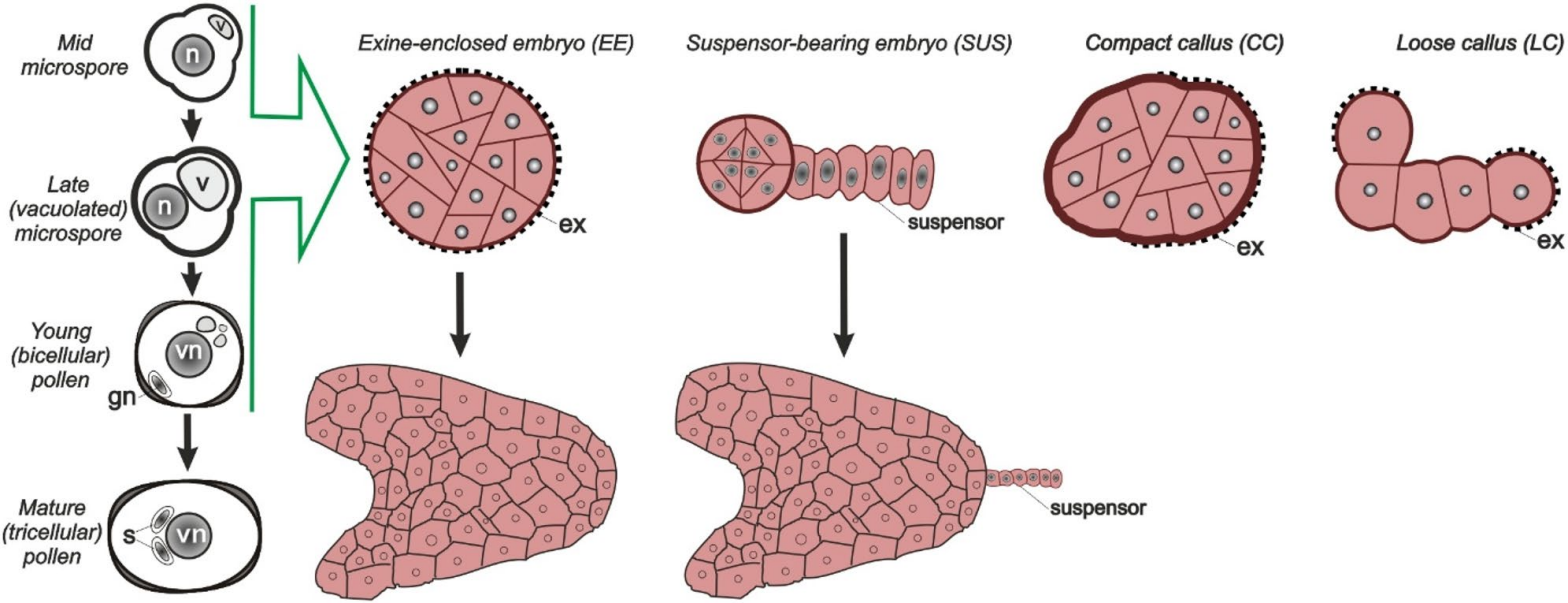
Developmental response of isolated *Brassica napus* microspores and pollen in culture. Cell cultures made from a mixture of microspores and young pollen grains are most responsive for haploid induction in vitro. Embryogenic structures can be identified by expression of zygotic embryo reporters (Soriano et al. 2014; Li et al. 2014). Four main types of embryogenic structures can be distinguished five days after the stress induction treatment: (1) exine-enclosed embryos (EE), (2) suspensor-bearing embryos (SUS), and two types of callus, (3) compact callus (CC) and (4) loose callus (LC). Time-lapse imaging of embryogenic structures from cultures treated with heat stress (HS, 33 °C) + 0.05 µM TSA (HS + TSA_low_) showed that these embryogenic structures differ in the plane of the first embryogenic division, the timing and extent of exine rupture, their cell wall composition and level of cellular adhesion, and in their ability to develop further into histodifferentiated embryos (Corral-Martínez et al. 2020; Siemons et al. 2025). *ex* exine, *n* microspore nucleus, *gn* generative nucleus, *vn* vegetative nucleus, *v* vacuole, *s* sperm cell

*Brassica napus* is a model system used to study ME. In this system, embryogenesis is induced by heat stress (HS): donor plants are grown at a low temperature (ca. 10 °C) followed by culture of isolated microspores/immature pollen (hereafter referred to collectively as microspores) at ca. 33 °C. Only a small fraction of the HS-treated microspores is competent to develop into a histodifferentiated embryo. In *B. napus*, four different types of multicellular embryogenic structures initially develop after the HS treatment (Fig. 1), each of which has a different capacity to develop into a histodifferentiated embryo (Li et al. 2014; Corral-Martínez et al. 2020): (1) exine-enclosed embryos (EE) that lack a suspensor, (2) suspensor-bearing embryos (SUS), and two types of embryogenic callus, (3) compact callus (CC) and (4) loose callus (LC). These structures are defined by differences in the first embryogenic cell division plane, the degree of exine rupture and intercellular adhesion, and by specific cell wall characteristics and composition (Corral-Martínez et al. 2020; Camacho-Fernández et al. 2021, 2024). Time-lapse imaging showed that exine-enclosed and suspensor embryos have the highest ability to survive (viability) and the greatest potential to form differentiated embryos, while compact and loose calli have the lowest viability, and of these, only loose calli occasionally develop into suspensor embryos (Corral-Martínez et al. 2020; Siemons et al. 2025).

Application of the histone/lysine deacetylase (HDAC/KDAC) inhibitor trichostatin A (TSA) in combination with HS enhances the induction of these embryogenic structures in a concentration and genotype-dependent manner. In the *B. napus* DH12075 genotype, the standard HS induction treatment produces very few embryos. Compared to the HS treatment, treatment with HS and a relatively low TSA concentration greatly enhances embryogenesis induction and embryo production, while treatment with HS and a relatively high TSA concentration increases embryogenesis induction to even higher levels but blocks the progression of embryo development (Li et al. 2014; Corral-Martínez et al. 2020).

Efficient ME depends on the combination of many different endogenous and exogenous factors. Previously, we showed that during ME, cytoplasmic cleaning is thought to facilitate embryogenesis by removing damaged molecules and cellular components that otherwise accumulate in large vacuoles that hinder cell division (Corral-Martínez et al. 2013). Additional factors, like the levels of cell wall callose and pectin methylesterase, and intracellular calcium levels, have also been shown to be relevant for embryo induction (Rivas-Sendra et al. 2017, 2019; Calabuig-Serna et al. 2024, 2025). Here we show that the competence of the different types of embryogenic structures for differentiated embryo formation is correlated with an ER stress response. ER stress is induced by many abiotic stresses and occurs when the normal folding and secretion capacity of the ER is exceeded (Pastor-Cantizano et al. 2020). ER stress initiates a highly conserved response called the unfolded protein response (UPR) that aims to refold misfolded or unfolded proteins to restore the ER’s folding and secretion capacity. Plants finely tune this response to balance growth and stress resilience (Ko and Brandizzi 2024).We observed that embryogenic structures with a low potential to complete embryo development showed stronger ER stress responses than those with a high potential to complete embryo development. The ER stress response was also dependent on the embryo induction treatment, with a combined HS and TSA treatment inducing a stronger response in all types of embryogenic structures than the HS treatment alone. Pharmacological modulation of ER stress response demonstrated that reduction of ER stress enhanced differentiated embryo formation. Together, these data suggest that recalcitrance for *B. napus* microspore-derived embryo development relies, in part, on the inability of embryogenic cells to restore cellular homeostasis after exposure to stress.

## Materials and methods

### Microspore-derived embryo culture

The *B. napus* low response genotype DH12075 was used for all experiments. Donor plant growth conditions and microspore isolation were performed as previously described (Soriano et al. 2014; Li et al. 2014). Briefly, donor plants were grown at 18 °C until bolting and then transferred to 10 °C. Populations of mid- to late uninucleate microspores (40,000 cells/ml) were used for pollen cultures or microspore-derived embryo cultures. To promote continued pollen development, isolated microspores were cultured for five days in NLN-13 medium at 18 °C (Custers et al. 1994). To induce embryogenesis, isolated microspores were cultured in NLN-13 medium for 24 h at 33 °C (HS), for 24 h with HS + 0.05 µM TSA (‘HS + TSA_low_’) or for 24 h with HS + 0.5 µM TSA (‘HS + TSA_high_’) followed by medium refreshment and further culture at 25 °C (Corral-Martínez et al. 2020).. TSA was dissolved in DMSO. The same volume of DMSO was added to the pollen and HS cultures.

The 4-phentylbutyrate (4-PB; 500 µM) stock solution was made in water, and the tunicamycin (Tm; 1 and 100 uM) stock solutions in DMSO. The stock solutions were filter-sterilized and stored at –20 °C. The required volumes of each stock solution were added to the microspore-derived embryo cultures to reach the desired final working concentrations (Tm: 0.001, 0.1 and 0.3 µM; 4-PB: 0.5, 5 and 50 µM). The same volume of DMSO (solvent) was added to the untreated controls. The compounds were applied with or without TSA for 24 h. The culture medium was refreshed after 24 h, and the compounds were added again to the medium. For each chemical tested, at least three biological replicates (1 ml culture; 40,000 cells/ml) were performed with three technical replicates per biological replicate. The effect of each compound was expressed as the number of embryos/ml of culture, assayed 14 days after the start of culture. All treatments were compared to each other in pairs using Student’s *t*-test, *p* ≤ 0.05. Treatments that do not statistically significantly differ from each other are labelled with the same letter.

Depending on the experiment, pollen or microspore-derived embryo cultures were analyzed at day 3, 4, 5, or 14 as indicated below. All compounds were obtained from Sigma-Aldrich.

For comparisons of embryo yield between treatments, statistical significance was evaluated using pairwise Student’s *t*-tests (two-tailed, *P* ≤ 0.05). Different letters above the bars indicate groups that do not differ significantly from each other. All statistical analyses were performed using SPSS Statistics (IBM Corp).

### Transmission electron microscopy

For transmission electron microscopy (TEM), samples from five-day-old HS, HS + TSA_low_ or HS + TSA_high_-treated microspore-derived embryos were recovered from in vitro culture dishes and processed as described in Corral-Martínez et al. (2011). Briefly, samples were fixed in Karnovsky fixative, post-fixed in 2% OsO_4_, dehydrated in an ethanol series, and finally embedded and polymerized in Embed 812 resin (Electron Microscopy Sciences). Samples were then sectioned (80–100 nm section thickness) with a Leica UC6 ultratome, mounted onto 200-mesh formvar and carbon-coated copper grids (Electron Microscopy Sciences), counterstained with uranyl acetate in 70% methanol (6 min) and lead citrate (30 s), and observed and imaged in a Philips CM10 transmission electron microscope. The ER and whole cell area of the pollen, exine-enclosed embryos, and embryogenic calli found in these cultures was measured as described in Jouannic et al. (2001). Areas were estimated by superimposing a single lattice test system (15 × 15 mm square size) over 15–20 randomly selected micrographs of each cell type taken at 60,000 x. Cells with ‘massive ER accumulation’ were defined as cells in which > 15% of the total cell area was occupied by ER. Lytic compartment accumulation in the cytoplasm and apoplast was scored manually. Three blocks were used for each sample and at least 50 images were analyzed per block.

Pairwise Fisher’s exact tests (*P* ≤ 0.05) were performed for comparisons of the frequency of massive ER accumulation (> 15% of cell area) between structure types under each treatment.

### Whole mount staining

Endoplasmic reticulum (ER) accumulation was observed using 3-dihexyloxacarbocyanine (DiOC_6_(3); Thermofisher), ER-Tracker Green (ThermoFisher) or the Cytopainter ER Staining Kit—Green Fluorescence (Abcam) staining and confocal laser scanning microscopy (CLSM).

For DiOC_6_(3) staining, samples from pollen developing *in planta*, from four-day-old pollen cultures (18 °C) and from four- or eight-day-old microspore-derived embryo cultures (HS, HS + TSA_low_, HS + TSA_high_) were first fixed overnight at 4 °C with 4% paraformaldehyde in PBS (pH 7.4). Samples were then washed three times with PBS and stored at 4 °C in 0.1% paraformaldehyde in PBS until use. Fixed samples were first stained with the nuclear stain 4′,6-diamidino-2-phenylindole (DAPI) as described in Rivas-Sendra et al. (2019), and then with 50 µM 3DiOC_6_(3) in PBS for 1 min. DiOC_6_(3) was excited with a 488 nm laser line and emission recorded between 504 and 510 nm.

For ER-Tracker Green (Thermo Fisher Scientific) staining, a 1 µM staining solution was prepared in PBS from a 1 mM DMSO stock and applied to fresh, live samples from four-day-old HS, HS + TSA_low_ and HS + TSA_high_-treated microspore-derived embryo cultures for 30 min at 25 °C. After staining, cells were fixed with 4% formaldehyde for 2 min and washed twice with PBS before imaging. Nuclear counterstaining with propidium iodide (PI) (10 µg/mL in PBS for 5 min) was performed prior to observation. Samples were analyzed using CLSM with excitation at 504 nm and emission at 511 nm for the ER-Tracker signal, and excitation at 535 nm and emission at 617 nm for PI.

For the Cytopainter ER (Abcam) staining, samples from four-day-old HS, HS + TSA_low_ and HS + TSA_high_-treated microspore-derived embryo cultures were fixed for 10 min in 3.7% paraformaldehyde and permeabilized with 0.1% Triton X-100 for 1 min. Cells were then stained with a solution containing 1 µL Green Detection Reagent and DAPI (1 µg/mL) in 1X Assay Buffer. The samples were incubated at 25 °C for 30 min. After washing with PBS, samples were analyzed by CLSM using excitation at 488 nm and emission at 510 nm for CytoPainter ER, and excitation at 358 nm and emission at 461 nm for DAPI.

All incubations were performed in the dark. Fluorescence was observed using a Leica DM5500 confocal microscope. Images were processed using the Leica Application Suite for Advanced Fluorescence software (LAS AF 2.7.3.9723).

All confocal microscopy images were acquired using identical imaging parameters (laser excitation power, detector gain, offset, zoom factor, scanning speed, and image resolution). At least three independent biological replicates were imaged per treatment and developmental structure type, with approximately 10–12 images recorded per replicate. Qualitative evaluation of ER staining was performed using approximately 100–120 embryogenic structures per treatment and developmental subtype in the HS + TSA and in vitro pollen cultures. Due to their inherently lower frequency, embryogenic structures in HS cultures were evaluated with approximately 30–40 structures per treatment and subtype.

### Transcriptome analysis

Three samples were used for transcriptome analysis: enriched embryogenic structures from 3-day-old HS + TSA_low_- and HS + TSA_high_-treated microspore-derived embryo cultures and three-day-old in vitro cultured pollen samples (18 °C).

Fluorescence activated cell sorting (FACS) of 3-day old HS + TSA_low_- and HS + TSA_high_-treated microspore-derived embryo cultures expressing the *LEC1:LEC1-GFP* embryo identity reporter (Li et al. 2014; Corral-Martínez et al. 2020; Siemons et al. 2025) was used to generate an enriched population of multicellular embryogenic structures for RNA-seq analysis. Immediately before sorting, the cultures were concentrated to 2.0 × 10^6^–4.0 × 10^6^ microspores/ml. Cultures were then filtered through 70-µm cell strainers to remove cell debris and kept on ice prior to FACS. Sorting was performed on a FACSAria II (BD Biosciences, San Jose, USA) equipped with a 100 µm nozzle, using PBS (pH 7.4) as sheath fluid (20 psi; 5000–10000 events s⁻^1^). Upon excitation at 488 nm, GFP-expressing embryogenic structures were selected based on fluorescence emission in the green channel (530 nm/30 nm band-pass filter) compared with wild-type negative controls. In the corresponding FACSDiva™ software, the emission signal of populations was visualized by PE-TEXAS RED-A set against GFP + YFP-A in a dot plot. The GFP-positive population of embryogenic cells was sorted directly into RNA extraction buffer (10,000 cells/100 uL buffer), then flash frozen in liquid N2 and stored at -80 °C.

Independent in vitro cultured pollen samples (18 °C) or FACS-sorted GFP positive samples from HS + TSA_low_- and HS + TSA_high_-treated *LEC1:LEC1-GFP* microspore-derived embryo cultures were pooled over one column to obtain a total of 150,000 sorted cells per biological replicate. RNA was purified using the Arcturus PicoPure RNA Isolation Kit (Thermo Fisher). For each replicate, ~ 10 ng of RNA was reverse-transcribed and amplified (12 PCR cycles) using the SMART-seq v4 Ultra-Low-Input kit (Takara Bio USA, Inc.). Amplified cDNA was then purified by immobilization on AMPure XP beads (Beckman Coulter) and the cDNA concentration was measured with a Qubit Fluorometer and the dsDNA HS Assay Kit (Thermo Fisher). The cDNA size distribution was checked using a 2100 Bioanalyzer and high sensitivity DNA kit following the manufacturer’s directions (Agilent).

The Nextera XT DNA Library Preparation Kit and the Nextera Index Kit (Illumina, 24 indexes, 96 samples, FC-131-1001) were used to prepare cDNA libraries according to the manufacturer’s instructions. As input, 0.125 ng amplified cDNA was used for the tagmentation reaction and 12 cycles for the enrichment PCR of adapter-ligated fragments. Clean-up of the cDNA libraries was performed using a ratio of 0.6 × volume of AMPure XP beads (Beckman Coulter). The cDNA library quality was checked as described above (typical libraries showed a broad size distribution of ~ 250–1000 bp). When needed, a second round of purification was performed using 0.8 × volume of cDNA:AMPure XP beads and SPRIselect beads (Beckman Coulter) for size selection. Libraries were diluted and sequenced with paired-end 150 base mode on a Novaseq S4 (Novogene Corporation Inc.).

Raw paired-end reads (125 bp) were quality-checked using FastQC (version 0.11.9) with default settings. Reads were aligned to the *B*. *napus* Darmor-bzh genome (version 10; https://www.genoscope.cns.fr/externe/plants/chromosomes.html) (Rousseau-Gueutin et al. 2020) using STAR (version 2.5.2b) (Dobin et al. 2012) with parameters for paired-end reads. Gene-level quantification was performed with featureCounts (version 2.0.2), (Liao et al. 2014) assigning mapped paired reads to annotated exons. DESeq2 (Love et al. 2014) was used to obtain normalized count values and to identify differentially expressed genes.

The RNA sequencing data can be retrieved from the Gene Expression Omnibus database under accession number GSE207576.

Heat maps generated in R function with pheatmap without clustering using log2(fold change) values.

### Ortholog annotation

Orthologous genes between *B. napus* Darmor-bzh v10 (BnaDAR) and arabidopsis were identified by integrating two lines of evidence: synteny information in the form of ancestral crucifer karyotype (ACK) blocks (He et al. 2021), and annotations from a *B. napus* pangenome (Song et al. 2020). A bidirectional all-versus-all sequence comparison between BnaDAR proteins and the arabidopsis reference proteome (UP000006548, UniProt) was constructed with DIAMOND v0.9.24 (Buchfink et al. 2014). First, a ‘synteny correction’ procedure was defined: each BnaDAR gene was initially assigned to the best-matching arabidopsis gene and its ACK block. Continuous runs of ACK block labels were identified in the BnaDAR annotations: for each ordered locus, a running sum of BIT scores is recorded for each ACK block label, and a decay rate of 0.9 is applied to all block scores at each step. The highest-scoring block is assigned as the ‘consensus block’ for this gene. If the consensus block for a gene did not match its initial label, the ortholog is non-syntenic. For each BnaDAR gene with a non-syntenic best hit, the highest-scoring hit belonging to the consensus block is chosen, if it exists.

To integrate BnaDAR genes with the *B. napus* pangenome, DIAMOND was used to align BnaDAR proteins against the proteomes of eight other *B. napus* accessions from Song et al. (2020). Each BnaDAR gene was assigned to the pangenome hub ID with the highest total BIT score of its members. The same procedure was followed to match arabidopsis gene IDs to *B. napus* hub IDs, and BnaDAR genes connected to the same hub ID are recorded as orthologs.

Where the two ortholog assignment methods disagree, these conflicts were resolved with the following decision tree: If only one method reported an ortholog, that ortholog is kept; if the methods disagreed and one of the reported orthologs is syntenic (matches the consensus ACK block), the syntenic ortholog is kept. For all other conflicts, default to the bidirectional best hit. The scripts used for pangenome assignment, synteny correction, and conflict resolution are available at https://github.com/maschon0/BnaDAR_synteny.

## Results

### ER expansion and protein-filled ER cisternae are ultrastructural signs of ER stress

**Fig. 2:**
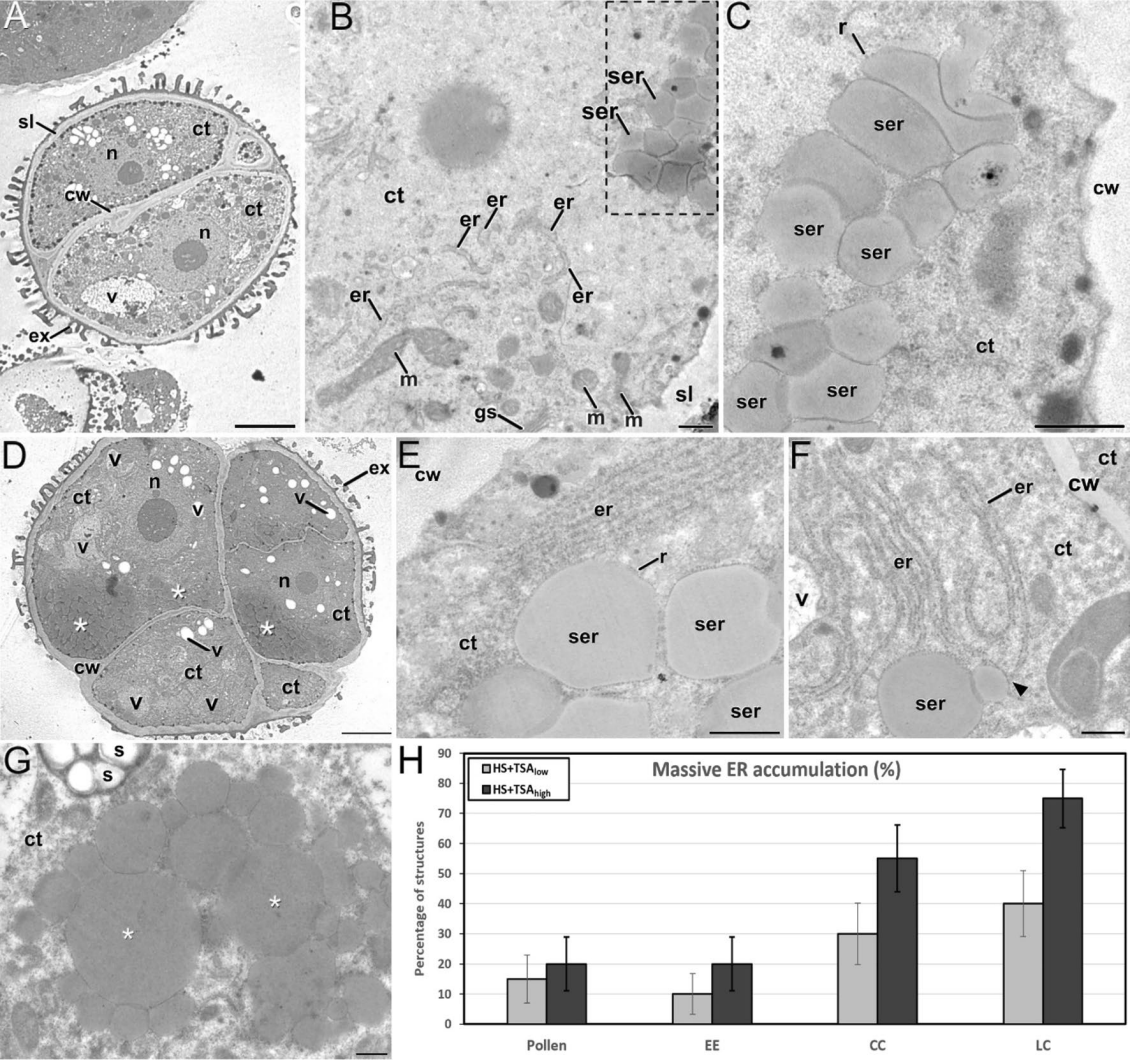
Ultrastructural analysis of embryogenic structures from 5-day-old HS + TSA_low_ microspore-derived embryo cultures. **A** Overview of an exine-enclosed embryo. **B** Region of the cytoplasm in an exine-enclosed embryo with abundant conventional ER cisternae (er) and swollen ER cisternae (ser) filled with electron-dense, likely proteinaceous materials (outlined box). **C** Detail of some of the swollen ER cisternae (ser) in the outlined box in B. Note their relatively uniform size, their electron-dense contents, and their rough contour, indicative of the presence of ribosomes (r) attached to the ER membrane. **D**. Overview of a compact callus. White asterisks indicate the presence of tightly packed, very electron-dense globules of variable size, concentrated at the bottom side of their respective cells, and surrounded by a ribosome-free membrane, as shown in (**G**). **E** Cytoplasmic region of a compact callus with stacked regular ER cisternae (er), as well as adjacent swollen ER cisternae (ser) filled with electron-dense materials and with ribosomes (r) attached to their membrane. **F** Detail of a region of a compact callus with regular and swollen ER cisternae (ser). A physical connection between the cisternae (arrowhead) is visible. **G** Detail of a compact callus showing a cluster of very electron-dense globules (white asterisks) of variable size that are surrounded by a ribosome-free membrane. **H** Proportion of the different types of structures in microspore-derived embryo cultures that show massive ER accumulation. Massive ER accumulation is defined as ER occupying > 15% of the cell area. The following structures were scored in 5-day-old microspore-derived embryo cultures induced with heat stress (HS) + 0.05 µM TSA (HS + TSA_low_) or HS + 0.5 µM TSA (HS + TSA_high_): pollen (non-embryogenic), exine-enclosed embryos (EE), compact calli (CC) and loose calli (LC). Error bars represent the standard error of the mean (SEM) of biological and technical replicates. Different letters indicate statistically significant differences between groups according to pairwise Fisher’s exact test (*P* ≤ 0.05). *ct* cytoplasm, *cw* cell wall, *er* endoplasmic reticulum, *ex* exine, *gs* golgi stack, *m* mitochondrion, *n* nucleus, *r* ribosome, *s *starch, *ser* swollen ER cisternae, *sl* subintinal layer, *v* vacuole. Bars in **A**, **D**: 5 µm; **B**, **C**, **E–G**: 500 nm

We performed transmission electron microscopy (TEM) to determine whether embryogenic structures show ultrastructural features of stress (Fig. 2). We examined the ultrastructure of exine-enclosed embryos and embryogenic calli from HS, HS + TSA_low_ (0.05 µM TSA) and HS + TSA_high_ (0.5 µM TSA)-treated cultures. Suspensor embryos could not be observed by TEM due to their very low abundance. In exine-enclosed embryos (Fig. 2A) from HS, HS + TSA_low_ and HS + TSA_high_-treated cultures, we observed ER membrane-enriched regions throughout the cytoplasm (Fig. 2B) and occasionally, adjacent to them, regularly sized deposits of electron-dense, likely proteinaceous material, surrounded by a membrane with attached ribosomes (Fig. 2C). The proximity of these membrane-bound deposits with regular ER cisternae, their contents, and the presence of ribosomes in the surrounding membrane suggested that these deposits are swollen, protein-filled ER cisternae. In embryogenic calli (Fig. 2D), we also observed the same type of ER-enriched regions and adjacent swollen ER cisternae (Fig. 2E). In some cases, the regular ER cisternae were tightly packed or even stacked (Fig. 2E). Clear physical connections could be observed between regular and swollen ER cisternae (Fig. 2F), further confirming their shared origin. These features—stacked rough ER, swollen protein-filled cisternae, and ER bodies—are classical ultrastructural markers of ER stress, as described for plant cells under both biotic and abiotic stress conditions (Bernales et al. 2006; Matsushima et al. 2003a,b). Finally, we also observed tightly packed, very electron-dense globules (heavily stained with lipid-binding OsO_4_) of variable size surrounded by a ribosome-free membrane (Fig. 2G). These clusters were always polarized and accumulated on one side in the different cells of the embryogenic structures (asterisks in Fig. 2D). Their polar localizations were most likely due to centrifugation. The variable size, osmophilic nature, absence of links with stacked ER, and the presence of a ribosome-free membrane in these clusters suggest that these are lipid droplets rather than protein-filled ER. Lipid droplets are thought to provide a non-toxic source of fatty acids for ER membrane and phagophore biogenesis and are also induced and degraded during autophagy for energy production and autophagic processes (Xu and Fan 2022).

To have a quantitative estimation of the level of ER expansion in the different microspore-derived structures, we used low magnification TEM images to quantify the relative proportion of pollen-like and embryogenic structures with massive ER accumulation (> 15% of the cell area) in HS + TSA_low_ and HS + TSA_high_-treated microspore-derived embryo cultures (Fig. 2H). The number of embryogenic structures in HS-treated cultures and suspensors in HS + TSA-treated cultures was too low to be counted in the TEM images. Statistically significant differences in massive ER accumulation were not detected between the different structures from HS + TSA_low_ cultures, while in HS + TSA_high_ cultures significant differences were observed in massive ER accumulation between structure types: compact calli (CC) and loose calli (LC) showed a markedly higher frequency of massive ER accumulation compared to exine-enclosed embryos (EE) and gametophytic structures (Fig. 2H). Of the 300 structures that showed massive ER accumulation in the HS + TSA_high_ treatment, ~ 70% were compact or loose calli, and only ~ 10% were exine-enclosed embryos and ~ 10% gametophytic structures. These results indicate that the enhancement of ER accumulation is structure-specific and is significantly pronounced only at the higher TSA concentration. Given that ER expansion and protein-filled ER are ultrastructural signs of ER stress, our data suggest that ER stress is more prevalent in embryogenic calli than in exine-enclosed embryos and pollen.

### ER stress is amplified in embryogenic structures with a low viability and reduced ability to form differentiated embryos

**Fig. 3:**
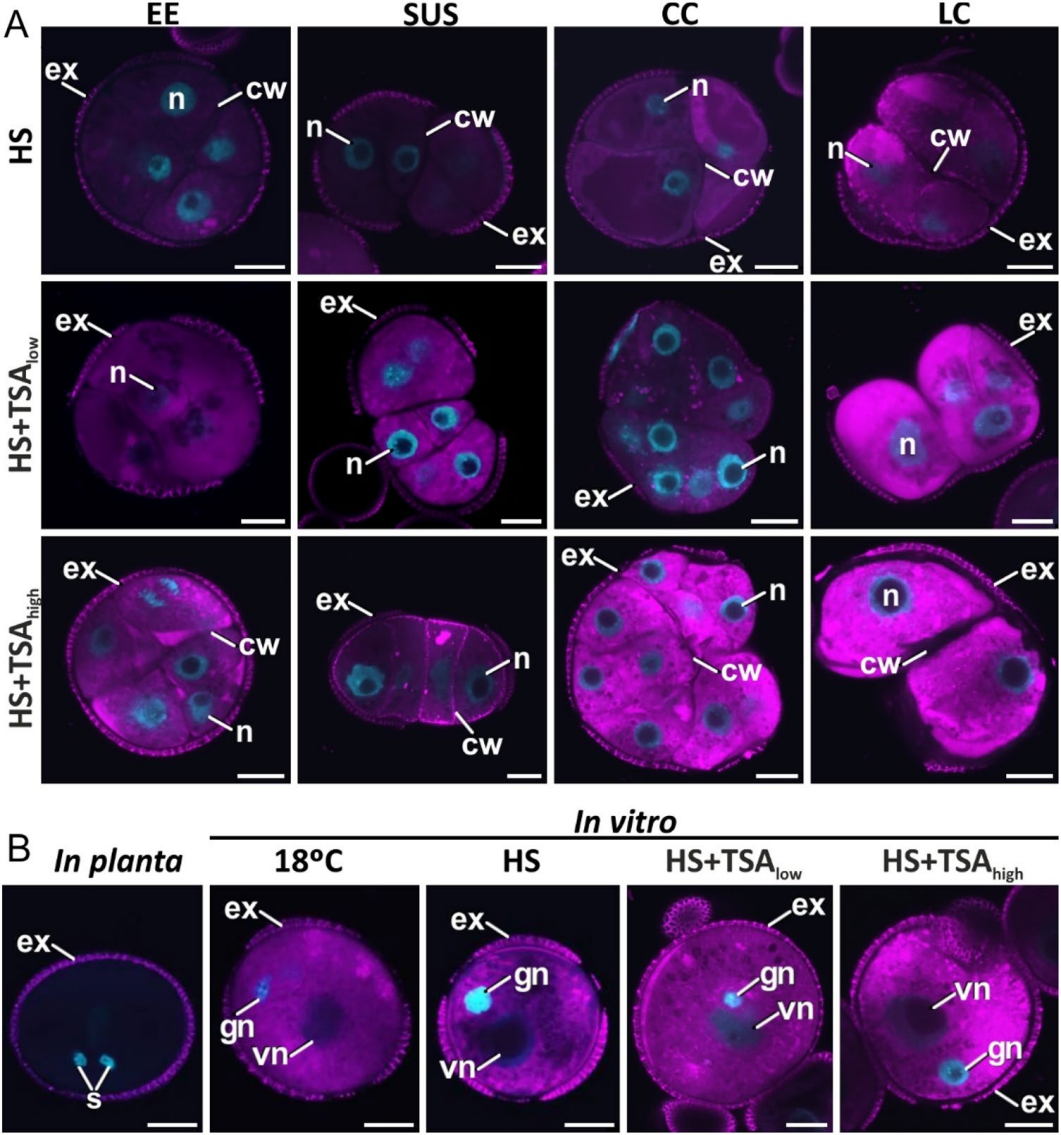
ER stress response is cell-type and treatment dependent. Confocal images of pollen and embryogenic structures stained with DiOC_6_ (magenta) and DAPI (blue). **A** ER stress response in the four different types of embryogenic structures found in 4-day-old microspore-derived embryo cultures is indicated at the top of the panels: EE (exine-enclosed embryos), SUS (suspensor-bearing embryos), CC (compact calli) and LC (loose calli). **B** ER stress response in pollen developing *in planta* and in vitro. Pollen was isolated directly from anthers (*in planta*), from four-day-old pollen cultures (18 °C) or from four-day-old microspore-derived embryo cultures induced by HS (heat stress, 33 °C); HS + TSA_low_ (HS + 0.05 µM TSA); and HS + TSA_high_ (HS + 0.5 µM TSA). *cw* cell wall, *ex* exine, *gn* generative nucleus, *n* nucleus, *s* sperm nucleus, *vn* vegetative nucleus. Bars: 10 µm

**Fig. 4:**
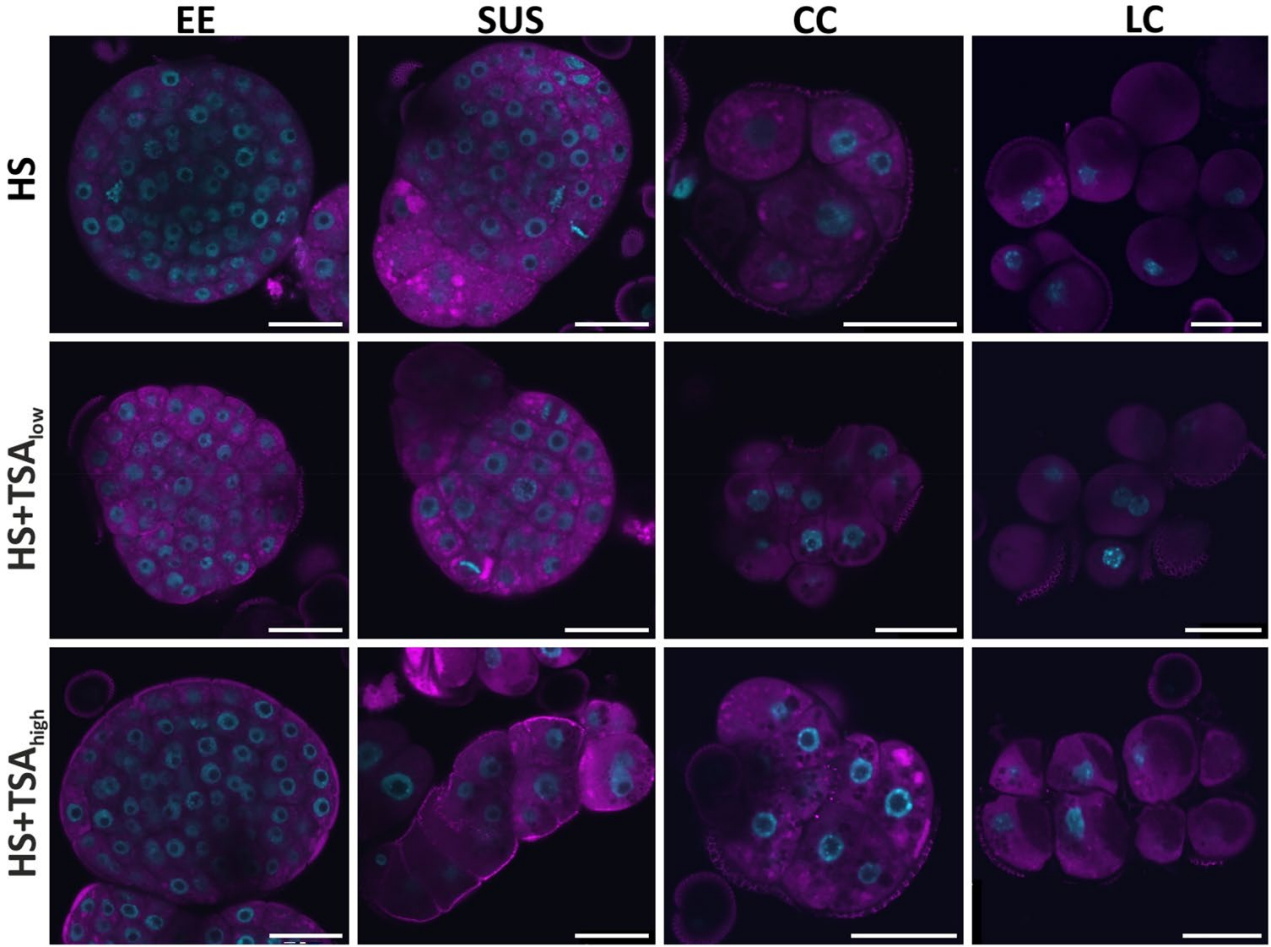
ER stress response in eight-day-old embryogenic structures from microspore-derived embryo cultures treated with HS (heat stress, 33 °C), HS + TSA_low_ (HS + 0.05 µM TSA), or HS + TSA_high_ (HS + 0.5 µM TSA). Confocal images of structures stained with DiOC_6_ (magenta) and DAPI (blue). The four different types of structures are indicated at the top of the panels: EE (exine-enclosed embryos), SUS (suspensor-bearing embryos), CC (compact calli) and LC (loose calli). Bars: 25 µm

We examined whether ER stress response is associated with the competence of microspores for ME. Staining with the DiOC_6_ internal membrane dye was used as a proxy to identify changes in ER accumulation in the four different embryogenic structures under different ME culture regimes, which allowed us to analyze a larger number of samples, including embryogenic structures in HS-treated cultures, and suspensor embryos in HS + TSA-treated cultures (Fig. 3). In four-day-old HS-induced microspore-derived embryo cultures, embryogenic structures with a low potential to form differentiated embryos (embryogenic calli) showed higher ER stress levels than exine-enclosed and suspensor-bearing embryos, both of which have a high potential to develop into differentiated embryos (Fig. 3A). In cultures treated with HS + TSA, DiOC_6_ staining increased in all types of embryogenic structures but was even higher in embryogenic calli (Fig. 3A). The staining patterns in the embryogenic structures were confirmed using two additional ER-specific stains, Cytopainter ER staining and ER-Tracker Green (Suppl. Fig. S1). Pollen developing *in planta* (before culture) showed very weak DiOC_6_ staining, if any (Fig. 3B). However, pollen cultured at non-embryo inducing conditions (18 °C), as well as the pollen-like structures that also develop in HS and HS + TSA-treated cultures, showed increased, clearly visible DiOC_6_ staining (Fig. 3B). As opposed to four-day-old embryogenic structures, where clear differences in DiOC_6_ staining were observed among structure types, in eight-day-old cultures (Fig. 4), i.e., seven days after cessation of the HS and TSA treatments, DiOC_6_ staining was generally faint, uniform and similar in all embryogenic structures, irrespective of the treatment or the type of structure.

Together, this data suggests that in vitro culture induces ER accumulation in few-celled embryogenic and pollen-like structures, and that TSA treatment further increases ER accumulation in these structures. ER accumulation is amplified in embryogenic structures with a low potential to survive and to form differentiated embryos (embryogenic callus) compared to those with a high potential to survive and form differentiated embryos (exine-enclosed and suspensor embryos). Later, the similar DiOC6 staining levels of all embryogenic structures after 8 days of culture suggest that these structures no longer show differential ER stress responses.

### Reducing ER stress improves haploid embryo production

We examined the effect of ER stress-modulating compounds on ER stress levels in embryogenic structures (measured by DiOC_6_ staining), as well as the effect of these compounds on the formation of differentiated embryos after two weeks of culture.

**Fig. 5:**
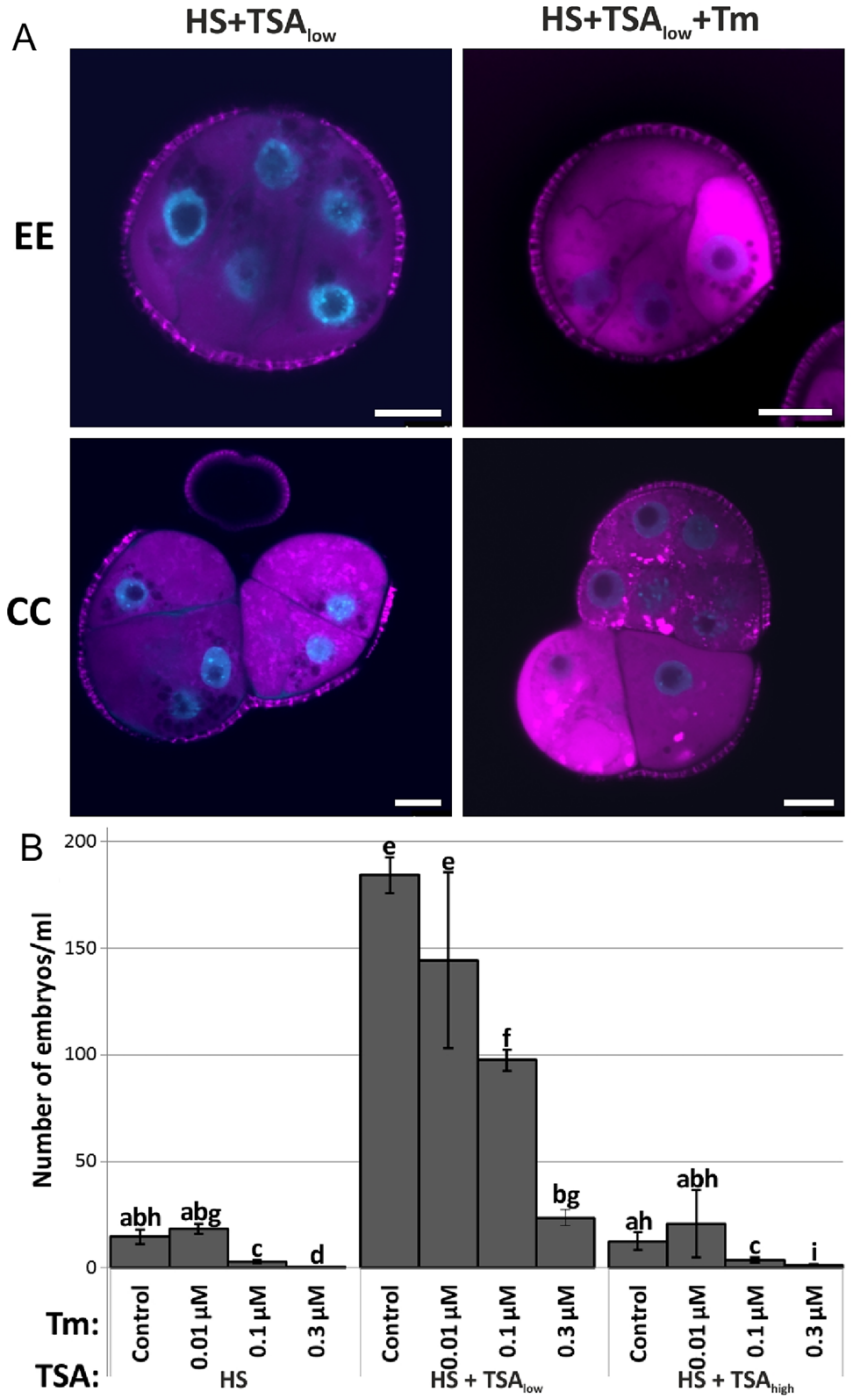
ER stress inhibits microspore embryogenesis. **A** Confocal images of Di0C_6_ (magenta) and DAPI (blue) staining of embryogenic structures (exine-enclosed embryos (EE) and compact callus (CC)) from 5-day-old HS + TSA_low_ (HS + 0.05 µM TSA) treated microspore-derived embryo cultures, treated with or without tunicamycin (Tm). Bars: 10 µm. **B** Embryo yield in cultures treated with or without (control) Tm at the indicated concentration in combination with the different induction treatments: HS (heat stress, 33 °C), HS + TSA_low_ (HS + 0.05 µM TSA) and HS + TSA_high_ (HS + 0.5 µM TSA). Embryo yield was assessed 14 days after the start of culture.Error bars represent the standard error of the mean (SEM) of biological and technical replicates. Treatments with the same letters are not significantly different according to pairwise Student’s *t*-tests (*P* ≤ 0.05)

Tunicamycin (Tm) inhibits biosynthesis of N-linked glycans, resulting in the formation of misfolded proteins in the ER and the concomitant induction of ER stress (Helenius and Aebi 2004). Continuous treatment with Tm increased the levels of ER stress in all types of structures (Fig. 5A) and decreased embryo yield in HS, HS + TSA_low_ and HS + TSA_high_-treated microspore-derived embryo cultures (Fig. 5B).

**Fig. 6:**
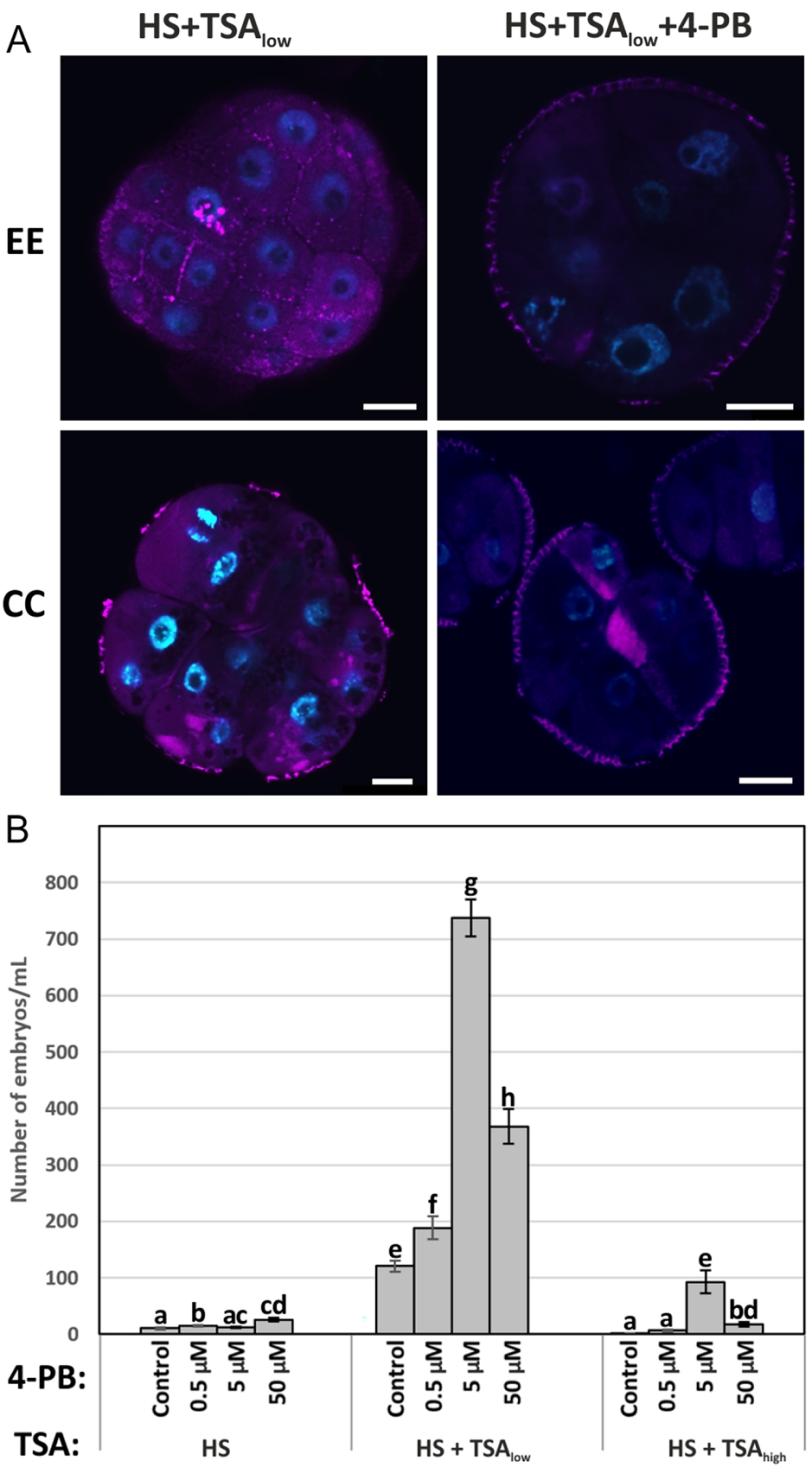
Relieving ER stress promotes microspore embryogenesis. **A** Confocal images of Di0C_6_ (magenta) and DAPI (blue) staining of embryogenic structures (exine-enclosed embryos (EE) and compact callus (CC)) from five-day old HS + TSA_low_ (HS + 0.05 µM TSA) microspore-derived embryo cultures, treated with or without 4-phenylbutyrate (4-PB). Bars: 10 µm.** B** Embryo yield of microspore-derived embryo cultures treated continuously without (control) or with the indicated 4-PB concentration in combination with the different induction treatments: HS (heat stress, 33 °C), HS + TSA_low_ (HS + 0.05 µM TSA), and HS + TSA_high_ (HS + 0.5 µM TSA). Embryo yield was assessed 14 days after the start of culture. Error bars represent the standard error of biological and technical replicates. Error bars represent the standard error of the mean (SEM) of biological and technical replicates. Different letters indicate statistically significant differences between treatments according to pairwise Student’s *t*-tests (*P* ≤ 0.05)

4-phenylbutyrate (4-PB) is a chaperone that assists in folding, maturation, and trafficking of misfolded proteins to relieve ER stress (Papp and Csermely 2006). ER stress was reduced with 4-PB treatment (Fig. 6A) and increased differentiated embryo yield in HS, HS + TSA_low_ and HS + TSA_high_ microspore-derived embryo cultures (Fig. 6B).

The pharmacological treatments described above suggest that ER stress negatively affects the ability of embryogenic structures to develop further into differentiated embryos, and by contrast, that reducing ER stress by promoting the UPR promotes cell competence for differentiated embryo formation.

### ER stress transcriptomes of embryogenic structures and pollen

**Fig. 7:**
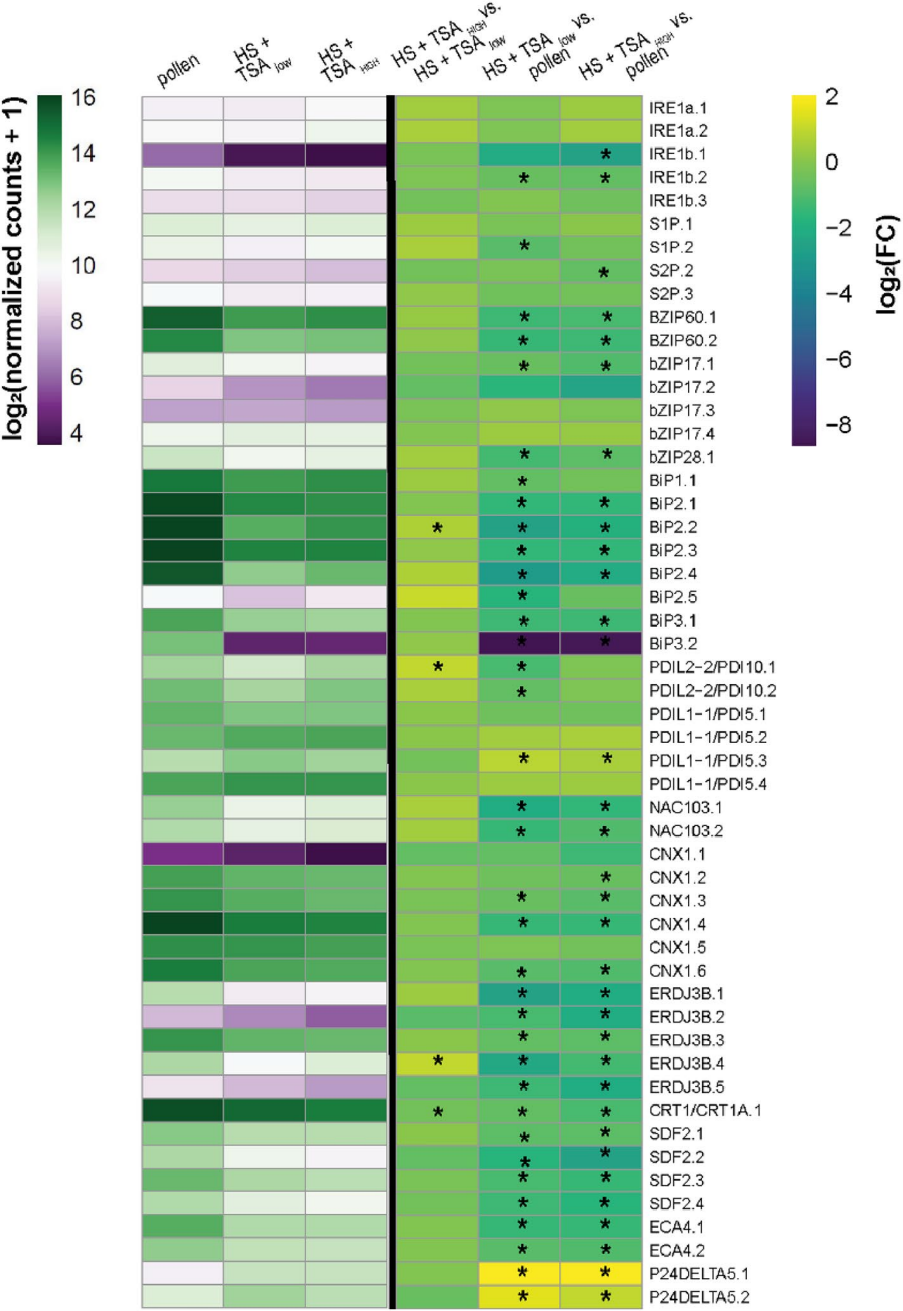
Transcriptional regulation of ER stress-related genes in embryogenic structures from microspore-derived embryo cultures and pollen cultures. The three left-most columns show a heat map of the normalized expression (log2 (normalized counts + 1)) of ER stress genes in FACs-purified embryogenic structures from three-day-old HS + TSA_low_ (HS + 0.05 µM TSA) and HS + TSA_high_ (HS + 0.5 µM TSA) microspore-derived embryo cultures and pollen from three-day-old 18 °C in vitro pollen cultures. The three right-most columns show the heatmap of the log2 fold change (log2(FC)) values for the pairwise comparisons HS + TSA_low_ and HS + TSA_high_, HS + TSA_low_ and pollen, and HS + TSA_high_ and pollen. The normalized gene expression scale bar is shown on the left and the gene expression ratio scale bar on the right. Gene expression ratios with an FDR value ≤ 0.05 are indicated with an asterisk (*). Co-orthologs of a given gene are numbered sequentially. The expression data is provided in Supplementary Data File S1

We used RNA-sequencing to compare how ER-stress-related genes are regulated at the transcriptional level in in vitro microspore-derived embryo cultures. Microspore-derived embryo cultures contain pollen at different developmental stages as well as the different embryogenic structures, the proportions of which depend on the culture conditions. To compare these two developmental pathways, we used Fluorescence-Activated Cell Sorting (FACS) to isolate a purified sample of embryogenic structures from three-day-old HS + TSA_low_ and HS + TSA_high_ microspore-derived embryo cultures and then compared these samples with samples from bulk three-day-old in vitro pollen cultures (18 °C). Both HS + TSA-treated samples comprise ca. 75% embryogenic calli and 25% exine-enclosed embryos (Corral-Martínez et al. 2020). The expression of the majority of ER stress genes known from arabidopsis for which we could identify a *B. napus* ortholog was down-regulated in HS + TSA-treated embryogenic structures compared to pollen (Fig. 7, Suppl. Data Set. S1). We did not observe dose-dependent down-regulation of gene expression between the two HS + TSA treatments, although there were a larger number of genes with statistically significant down-regulated expression in the HS + TSA_high_ versus HS + TSA_low_ treatment. Given that the majority of the embryogenic structures in the two samples are embryogenic calli, our data suggest that embryogenic calli are less competent than pollen to activate a transcriptional ER stress response.

## Discussion

### ER bodies are produced in stressed *B. napus* embryogenic microspores

In this work, we showed that embryogenic microspores are characterized by regions of stacked ER cisternae and expanded rough ER cisternae filled with proteins (Fig. 2). Both ultrastructural features are indicative of ER stress. These changes were inversely correlated with the competence of embryogenic structures for their further development into differentiated embryos and were also amplified in each type of embryogenic structure when HDAC activity was reduced using TSA.

Stacked ER has been described in animal cells as a mechanism to maximize the synthesis of secretory proteins (Terasaki et al. 2013). ER expansion and changes in ER morphology have been observed in response to ER stress in different organisms (Sriburi et al. 2004; Bernales et al. 2006; Schuck et al. 2009) including plants (Qiang et al. 2012). Heat stress is known to induce ER stress in plants (Deng et al. 2011; Li and Howell 2021; Singh et al. 2021), and there is increasing evidence of a role of the ER in stress-related processes such as autophagy (Zeng et al. 2019). We observed that stacked ER regions are connected to swollen rough ER cisternae filled with proteins (Fig. 2F) that strongly resemble ER bodies. ER bodies are round or elongated cisternae of rough ER filled with proteins (Bonnett and Newcomb 1965; Iversen 1970). ER bodies have only been found in the Cruciferae, where they are constitutively found in different tissues and also in response to biotic and abiotic stresses (Matsushima et al. 2003a, b). Although their precise function is still unknown, the identification of a β-glucosidase as a main component of *Arabidopsis thaliana* (arabidopsis) ER bodies led to the suggestion that ER bodies have a role in plant defense against insect attack or wounding (Matsushima et al. 2003c). In the context of ME, this would not be surprising, since production of AGPs, hordeins, chitinases, and other pathogen-related factors have been found in embryogenic microspores in *B. napus* and other species (Vrinten et al. 1999; Borderies et al. 2004; Pulido et al. 2006). β-Glucosidases are involved in other processes such as hormone activation and cell wall remodeling (Borderies et al. 2004; Cairns and Esen 2010). Interestingly, embryogenic microspores and multicellular embryogenic structures undergo profound cell wall remodeling, the extent of which determines their developmental fate (Camacho-Fernández et al. 2021, 2024) Thus, a role for the ER bodies found in *B. napus* embryogenic microspores in these processes should not be discarded.

In addition to β-glucosidase, ER bodies contain hormones, metabolites, and enzymes, including the precursors of two stress-inducible cysteine proteinases and a vacuolar processing enzyme (Hayashi et al. 2001; Matsushima et al. 2003b). When arabidopsis epidermal seedling cells are treated with high salt levels, the ER bodies fuse with each other and with vacuoles, thereby delivering enzyme precursors to the vacuoles. Formation of ER bodies could provide a system for proteinase storage under various stress conditions. This is consistent with the fact that ER bodies have not yet been observed in *B. napus* microspores in in vivo conditions, but we observed ER bodies in all in vitro embryogenic structures, and more abundantly in HS + TSA-treated structures (Camacho-Fernández et al. 2021, 2024).Thus, in the context of ME, formation of ER bodies might also provide a mechanism to increase proteolytic activity to deal with a large amount of mis- or unfolded proteins that occur in embryogenic cells in response to stress.

### ER stress limits cell totipotency

We observed differences in ER stress response among the different types of embryogenic structures that correlate with the ability of these structures to develop further into differentiated embryos. Currently, the only known differences between embryogenic structures with a high viability (exine-enclosed and suspensor embryos) and embryogenic structures with a low viability (calli) are their cell wall composition, degree of cellular adhesion, and extent of exine rupture. Exine-enclosed and suspensor embryos show higher cellular adhesion, controlled exine rupture, and outer cell walls enriched in callose, highly methyl-esterified pectin, and some types of arabinogalactan proteins (AGPs), while embryogenic calli have lower cellular adhesion, undergo sudden and massive exine rupture, and their outer cell walls show lower levels of these cell wall components (Corral-Martínez et al. 2013; Camacho-Fernández et al. 2021, 2024; Siemons et al. 2025). However, it is not known whether these specific features of embryogenic calli are a cause or consequence of their inability to resolve ER stress, as the different embryogenic pathways can only be distinguished morphologically after exine rupture. Time-lapse imaging with fluorescent ER stress and other abiotic stress reporter lines would help to resolve this point. Regardless, ER stress adds to the features that limit cell totipotency and prevent embryogenic microspores from developing into functional embryos.

### In vitro* pollen resolves ER stress response better than embryogenic calli*

ME is only induced in a small proportion of microspores; the remainder either stop dividing or continue pollen-like development (Seguí-Simarro and Nuez 2008). The ability of pollen to survive HS in culture is in stark contrast to the situation *in planta*, where pollen development is highly sensitive to acute HS (Müller and Rieu 2016). Compared to *B. napus* pollen developing *in planta, *in vitro cultured pollen showed enhanced ER synthesis (Fig. 3), yet mature (tricellular) pollen grains develop from these pollen and even germinate when cultured continuously under HS conditions (Custers et al. 1994). The ability of isolated pollen to develop further under HS conditions, together with the observation that ER stress genes are upregulated in pollen compared to embryogenic structures (mostly calli) (Fig. 7), suggests that in vitro pollen grains experience ER stress but are able to maintain their cellular equilibrium. Pollen germination requires high levels of secretory proteins and membrane trafficking for pollen tube growth, thus higher basal levels of UPR components in pollen (Fragkostefanakis et al. 2016) might help to resolve the increased levels of ER stress in tissue culture.

### HDAC regulation of ER stress response

Addition of acetyl groups to histone lysine residues by histone/lysine acetyl transferases and their removal by HDACs is a reversible mechanism to control gene expression. Histone acetylation is generally associated with transcriptional activation, whereas histone deacetylation is associated with transcriptional repression. We show that ER stress responses are enhanced after a combined HS + TSA treatment, generally in a dose and embryogenic structure-specific manner (Fig. 2, Fig. 3). These results suggest that HDACs not only regulate the competence of microspores for ME, but also their stress responses. However, we do not know whether ER stress responses are regulated directly by HDACs during ME. HDACs also deacetylate non-histone nuclear and cytoplasmic proteins (Füßl et al. 2018; Zheng et al. 2020; Xu et al. 2021), therefore, identification of the HDAC-regulated gene loci and/or proteins that contribute to the ER stress response during ME is an important topic for future research.

The HDACs that repress embryo identity in pollen cells are not known, but multiple HDAC proteins are likely involved as embryogenic divisions were not induced in pollen of single arabidopsis HDAC mutants in the absence of TSA (Li et al. 2014). HDAC proteins have broad functions throughout development, including regulation of abiotic stress response (Luo et al. 2017). TSA is a pan-inhibitor that targets Rpd3/Hda1, of which there are 12 genes in the arabidopsis genome (Pandey et al. 2002). Thus, TSA-induced activation of stress response and totipotency likely reflects the sum of the inhibition of different HDAC proteins. Moreover, individual HDAC proteins can have either agonistic or antagonistic relationships in different pathways; thus, the two processes, stress response and totipotency, might be regulated by the same or different HDAC proteins. For example, of the 12 arabidopsis HDAC proteins that are inhibited by TSA, HDA19 has been shown to negatively regulate HS response and HDAC6 and HDA9 to positively regulate HS response (Popova et al. 2013; Ueda et al. 2018; Niu et al. 2022), while HDA6 and HDA19 cooperatively repress totipotency in seedlings (Tanaka et al. 2008), and HDA19 restricts hormone-induced somatic embryogenesis (Morończyk et al. 2022). Single and higher-order HDAC mutant analysis in *B. napus* would help to identify the HDAC proteins responsible for promoting stress-induced ME.

## Conflict of interest

None.

## Supplementary Information

Below is the link to the electronic supplementary material.

Supplementary file1 (JPG 824 KB)

Supplementary file2 (XLSX 29 KB)

## Data Availability

The RNA sequencing data can be retrieved from the Gene Expression Omnibus database under accession number GSE207576. The scripts used for pangenome assignment, synteny correction, and conflict resolution are available at https://github.com/maschon0/BnaDAR_synteny.
