# Supplementary figures and images for: Unresolved ER stress restricts in vitro plant cell totipotency
Source: Plant Cell Rep. 2025 Sep 17;44(10):214. doi: 10.1007/s00299-025-03586-8 (PMC12443906; doi:10.1007/s00299-025-03586-8)

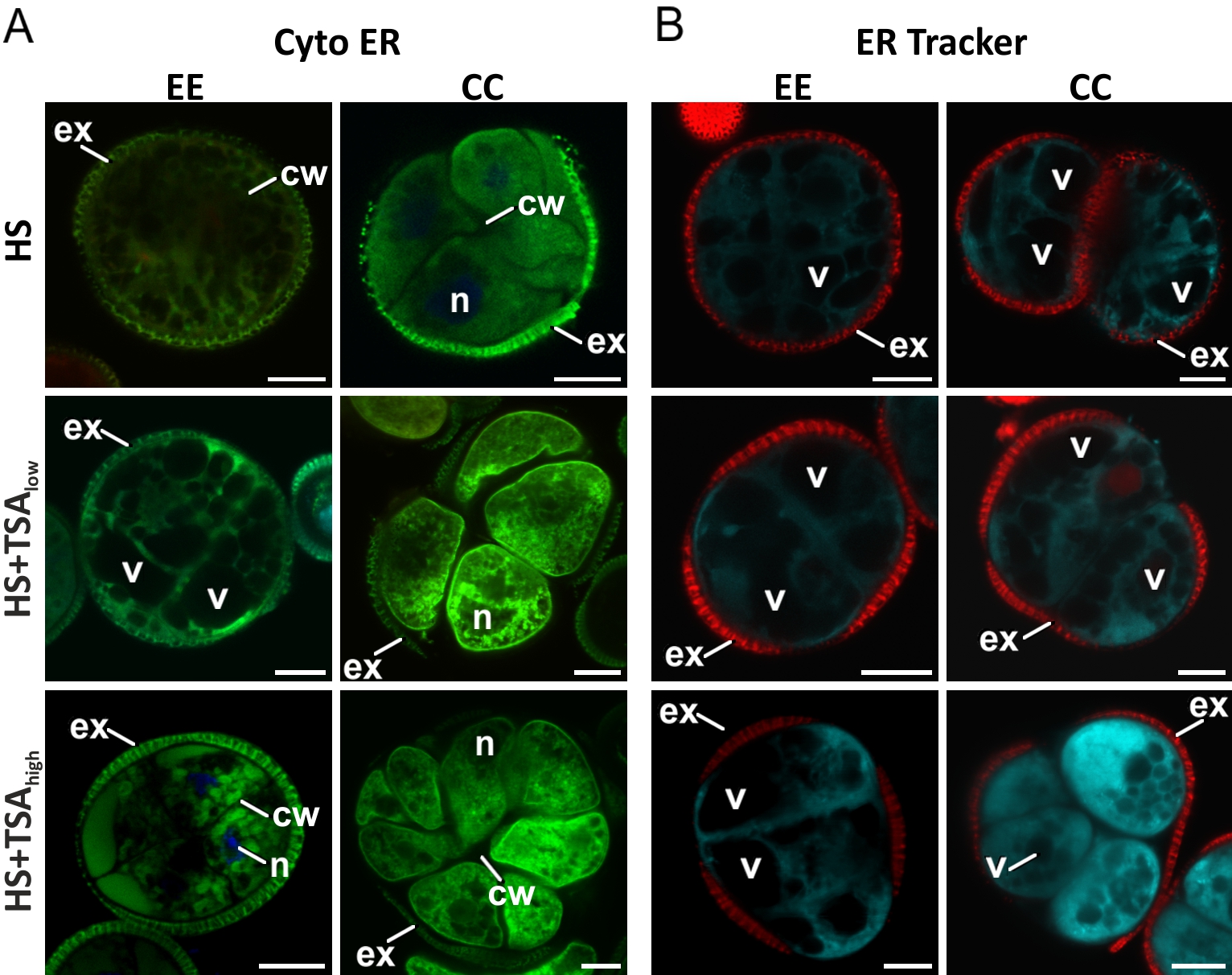

Supplement: Supplementary file 1 — Supplementary file1 (JPG 824 KB) [file 299_2025_3586_MOESM1_ESM.jpg]
